# Integrated information as a metric for group interaction

**DOI:** 10.1371/journal.pone.0205335

**Published:** 2018-10-11

**Authors:** David Engel, Thomas W. Malone

**Affiliations:** 1 Center for Collective Intelligence, Massachusetts Institute of Technology, Cambridge, Massachusetts, United States of America; 2 Sloan School of Management, Massachusetts Institute of Technology, Cambridge, Massachusetts, United States of America; Georgia Institute of Technology, UNITED STATES

## Abstract

Researchers in many disciplines have previously used a variety of mathematical techniques for analyzing group interactions. Here we use a new metric for this purpose, called “integrated information” or “phi.” Phi was originally developed by neuroscientists as a measure of consciousness in brains, but it captures, in a single mathematical quantity, two properties that are important in many other kinds of groups as well: differentiated information and integration. Here we apply this metric to the activity of three types of groups that involve people and computers. First, we find that 4-person work groups with higher measured phi perform a wide range of tasks more effectively, as measured by their collective intelligence. Next, we find that groups of Wikipedia editors with higher measured phi create higher quality articles. Last, we find that the measured phi of the collection of people and computers communicating on the Internet increased over a recent six-year period. Together, these results suggest that integrated information can be a useful way of characterizing a certain kind of interactional complexity that, at least sometimes, predicts group performance. In this sense, phi can be viewed as a potential metric of effective group collaboration.

## Introduction

A vast number of phenomena in the world arise out of the interactions of individuals in groups, from the emotional tone of a family [1,2] to the productivity of an economy [3] to the spread of disease in a community [4], and researchers in a variety of disciplines have used many different mathematical tools to analyze these phenomena. For instance, psychologists have used Markov models to analyze the sequences of actions in small groups of people [5–7], economists have used general equilibrium theory to analyze the interactions among buyers and sellers in a market [8], and sociologists have used graph theory to analyze various kinds of social networks [4,9].

In this paper, we examine another mathematical technique that has not previously been used for analyzing group interactions. This technique, based on information theory, is intriguing because it was developed as a physical measure that would quantify the level of consciousness of a brain [10–14]. We will see, however, that the metric is general enough to apply to many other kinds of systems, and we focus here on using it to analyze groups of people and computers.

### What is integrated information?

The metric we use is called “integrated information” or “phi” and was proposed by Tononi and colleagues [10–14]. There have been several successively refined versions of phi (summarized in [12]), but all the versions aim to quantify the integrated information in a system. Loosely speaking, this means the amount of information generated by the system as a whole that is more than just the sum of its parts. The phi metric does this by splitting the system into subsystems and then calculating how much information can be explained by looking at the system as a whole but not by looking at the subsystems separately.

In other words, for a system to have a high value of phi, it must, first of all, generate a large amount of *information*. Information can be defined as the reduction of uncertainty produced when one event occurs out of many possible events that might have occurred [15]. Thus, a system can produce more information when it can produce more possible events. This, in turn, is possible when it has more different parts that can be in more different combinations of states. In other words, a system needs a certain kind of differentiated complexity in its structure in order to generate a large amount of information.

But phi requires more than just information; it also requires the information to be *integrated* at the level of the system as a whole. A system with many different parts could produce a great deal of information, but if the different parts were completely independent of each other, then the information would not be integrated at all, and the value of phi would be 0. For a system to be integrated, the events in some parts of the system need to depend on events in other parts of the system. And the stronger and more widespread these interdependencies are, the greater the degree of integration.

For instance, a single photodiode that senses whether a scene is light or dark does not generate much information because it can only be in two possible states. But even a digital camera with a million photodiodes, which can discriminate among 2^1,000,000^ possible states, would not produce any integrated information because each photodiode is independently responding to a different tiny segment of the scene. Since there are no interdependencies among the different photodiodes, there is no integrated information [13].

Tononi and colleagues argue that these two properties—differentiated information and integration—are both essential to the subjective experience of consciousness. For example, the conscious perception of a red triangle is an integrated subjective experience that is more than the sum of perceiving “a triangle but no red, plus a red patch but no triangle” [12]. The information is integrated in the sense that we cannot consciously perceive the triangle’s shape independently from its color, nor can we perceive the left visual hemisphere independently from the right. Said differently, integrated information in conscious experience results from functionally specialized subsystems that interact significantly with each other [16].

Even though there is some empirical evidence that the mathematical behavior of phi is consistent with empirical observations of human consciousness (e.g., [10,17–21]), there is still considerable debate among researchers about whether phi actually measures consciousness (e.g., [22]). The most recent version of integrated information theory [12] also specifies additional requirements for a system to be conscious, such as the “exclusion” postulate which says that in nested systems, only the system at the level with the maximum value of phi can be conscious.

It is also important to note that in order to apply the theoretical definitions of phi, a complete model of the rules governing state transitions in the system is needed. Since such models are rarely available for observational data, however, we used here two alternative versions of phi suggested by Barrett and Seth [23]. These measures estimate conditional probabilities for state transitions from the actual observed data (see Methods). Later work has pointed out limitations of (and possible corrections for) these versions of phi as estimates of the original theoretical definitions of phi [24, 25]. But these definitions of phi still measure the two properties used in the original definitions of phi—differentiated information and integration.

And, interestingly, these two properties are similar to properties that are also important in many other kinds of systems. For example, Adam Smith [26] observed that economic systems are often more productive when (a) division of labor leads different people to specialize in different kinds of work and (b) the “invisible hand” of the market integrates their diverse efforts. Lawrence and Lorsch [27] discussed the importance of differentiation and integration in large, hierarchical human organizations: (a) dividing the organization into specialized subunits and (b) integrating these units to achieve the goals of the overall organization. And in many fields of engineering and other kinds of design, effective problem solving often involves (a) dividing a problem into subparts and (b) integrating solutions for the subparts into a solution for the whole problem [28–30].

In other words, the mathematical concept of integrated information provides a quantitative way of measuring a combination of two properties that are important across a wide range of different types of systems. And whether phi is measuring consciousness or not, it is clearly measuring something that is of potential interest to many different disciplines.

### A mathematical formulation of integrated information

The concept of integrated information, or phi, can be represented mathematically as follows [23]:

$$\emptyset =\sum_{k=1}^{r}H(M_{0}^{k}|M_{1}^{k})-H(X_{0}|X_{1}),$$ (1)

where H(X|Y) is the entropy of variable X given knowledge of variable Y, X_0_ and X_1_ are the states of the whole system at time t_0_ and t_1_, respectively, and <inline> and <inline> are subsets of X that completely partition the parts of X at these times. For example, <inline> quantifies how much of the uncertainty of subsystem k at time t_0_ cannot be explained by knowledge of the state of the subsystem at time t_1_.

Summing over all subsystems (the first term in Eq (1)) gives us the amount of entropy that cannot be explained by the subsystems themselves. The second term in Eq (1) quantifies the conditional entropy of the whole system. Thus phi is high if there is a large amount of entropy that cannot be explained by looking at the subsystems separately but that is explained by looking at the system as a whole.

The value calculated by Eq (1) is the phi as defined by Tononi and colleagues if and only if the partitioning is chosen as the minimum information bipartition (MIB), that is, the decomposition into two parts that are most independent. More thorough descriptions of phi can be found in [11–14,20,31].

### Interpreting differentiation and integration as integrated information

This mathematical formulation of phi is broadly consistent with the concepts of differentiation and integration described above. To see how, consider a simple example involving different ways of organizing the production of automobiles. In the early years of the 20^th^ century, there were hundreds of small automobile companies, each of which produced a few handmade cars [32]. Using the notation above, we can represent the state of one of these companies *k* at time *t* as $M_{t}^{k}$, with each element of $M_{t}^{k}$ representing whether a given part of the company (such as a person) is active at time *t*. (A more detailed representation could include a separate element for each type of activity the person could do at time *t*).

When there are more companies that contain more different elements, then system X can potentially generate more information. The amount of information the system actually generates, however, will depend on how differentiated the activities of the companies are. For instance, if many of the companies depend on the same suppliers for the same parts, then shortages of those parts will affect all the companies in a similar way. But if the companies use different parts and different suppliers, then more combinations of their states are possible, and the system as a whole generates more information.

Regardless of how differentiated the companies are, if the companies are not integrated, that is, if they operate relatively independently of each other, then almost all the entropy in the system can be explained by looking at each company separately. In this case, then, there would be very little integrated information at the level of the whole group of companies.

But if, instead of being organized as separate companies, all the participants in the whole system X were part of a single large company, then we might represent the state of each different part of the company as a different $M_{t}^{k}$. For instance, if we assume that the company is organized with different high-level functions (such as engineering, manufacturing, and sales), then we could consider each of these functions as a separate part.

In this case, the different parts of the company would be differentiated because they would be doing different kinds of activities. They would also be integrated because all of these functions have to be coordinated to produce even a single car. That means there would be substantial interdependencies among all the different parts of the company. For instance, a major delay in the engineering design for a new car could significantly delay the manufacturing and sales activities for that car. Thus, there would be substantial amounts of entropy that could be explained only by looking at the whole company, not by looking at each of the parts separately. And, thus, there would be substantial integrated information in this system.

Of course, increasing the differentiation and integration in a system does not always increase the system’s performance. But as we saw above, these two features figure prominently in a number of theories of group performance in human groups, so it is at least reasonable to hypothesize that they are correlated with performance. And there are also several simulation studies that suggest a relationship between performance and the combination of differentiation and integration that is measured by integrated information [31,33,34].

### Applying the phi metric

Since phi provides a quantitative measure that combines differentiation and integration, we first test whether it is, in fact, correlated with performance in two different kinds of groups: (a) small groups of experimental subjects working together on shared laboratory tasks, and (b) groups of Wikipedia contributors improving Wikipedia articles over time. As a further test of the applicability of phi, we also examine whether it detects what we might assume would be the increasingly differentiated and integrated complexity of the Internet over time. We evaluate this by applying the phi metric to data about all the computers (and people) communicating over a specific Internet backbone during a six-year period.

In order to apply the versions of phi we used [23], we needed to determine: (a) a characterization of the state of the system at different times, and (b) a time delay with respect to which phi will be calculated [10].

## Results

### Study 1: Small work groups

In Study 1, we applied the phi metric to interaction data we gathered from a previous study of groups performing a series of tasks designed to measure their collective intelligence [35]. Collective intelligence (CI) is a statistical factor for a group that predicts the group’s performance on a wide range of tasks, just as individual intelligence does for individuals [36]. Following [36], we measured collective intelligence by analyzing the performance of small work groups performing a range of diverse tasks (see Methods).

**Fig 1:**
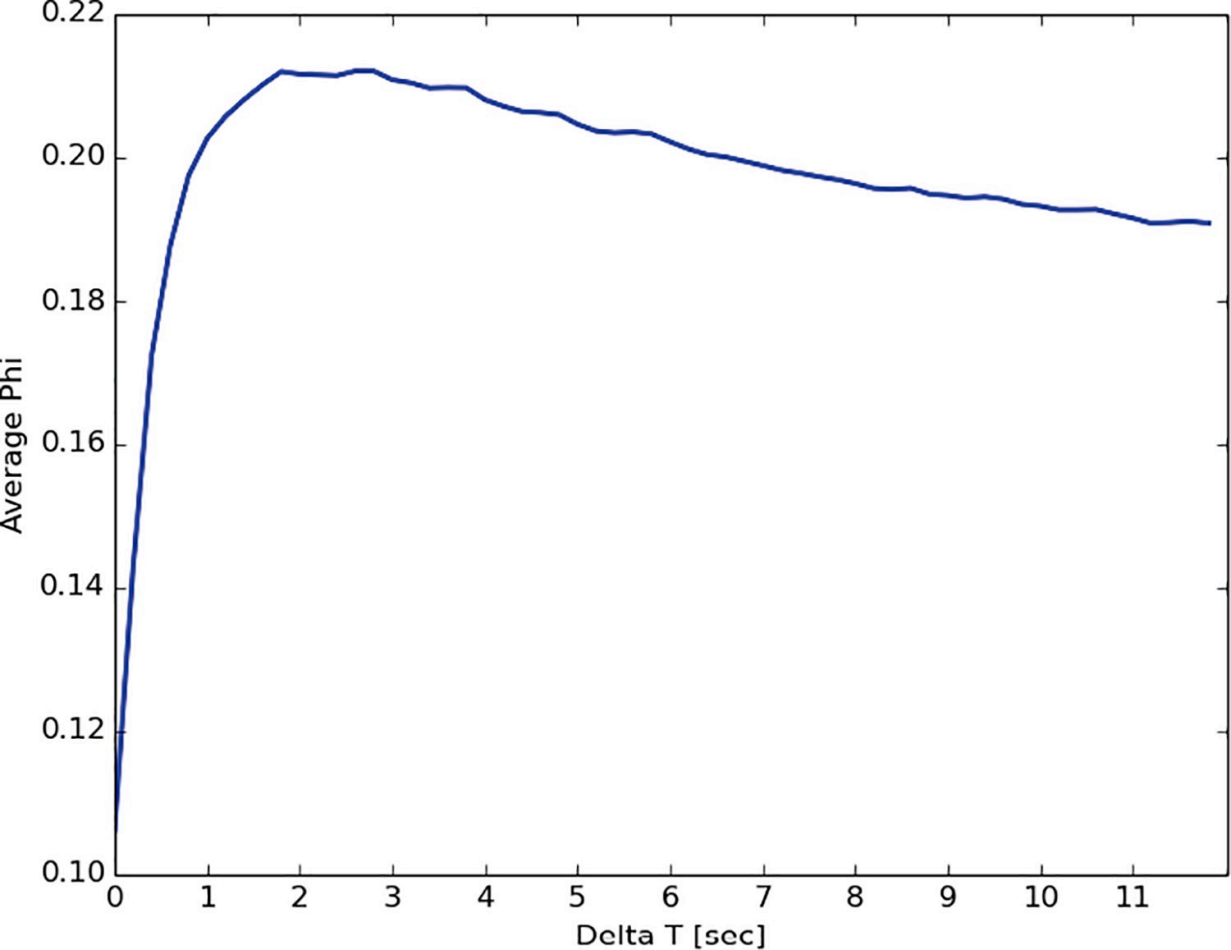
Average phi for face-to-face groups computed with different time delays.

In order to apply the phi metric, we characterized the state of the group in terms of which group member was communicating at which point in time (see Methods). To determine the time delay with respect to which phi should be calculated for face-to-face groups, we plotted average phi for different time delays (Fig 1). There is a clear peak at around 2 seconds, an intuitively plausible value, so we used this value.

**Fig 2:**
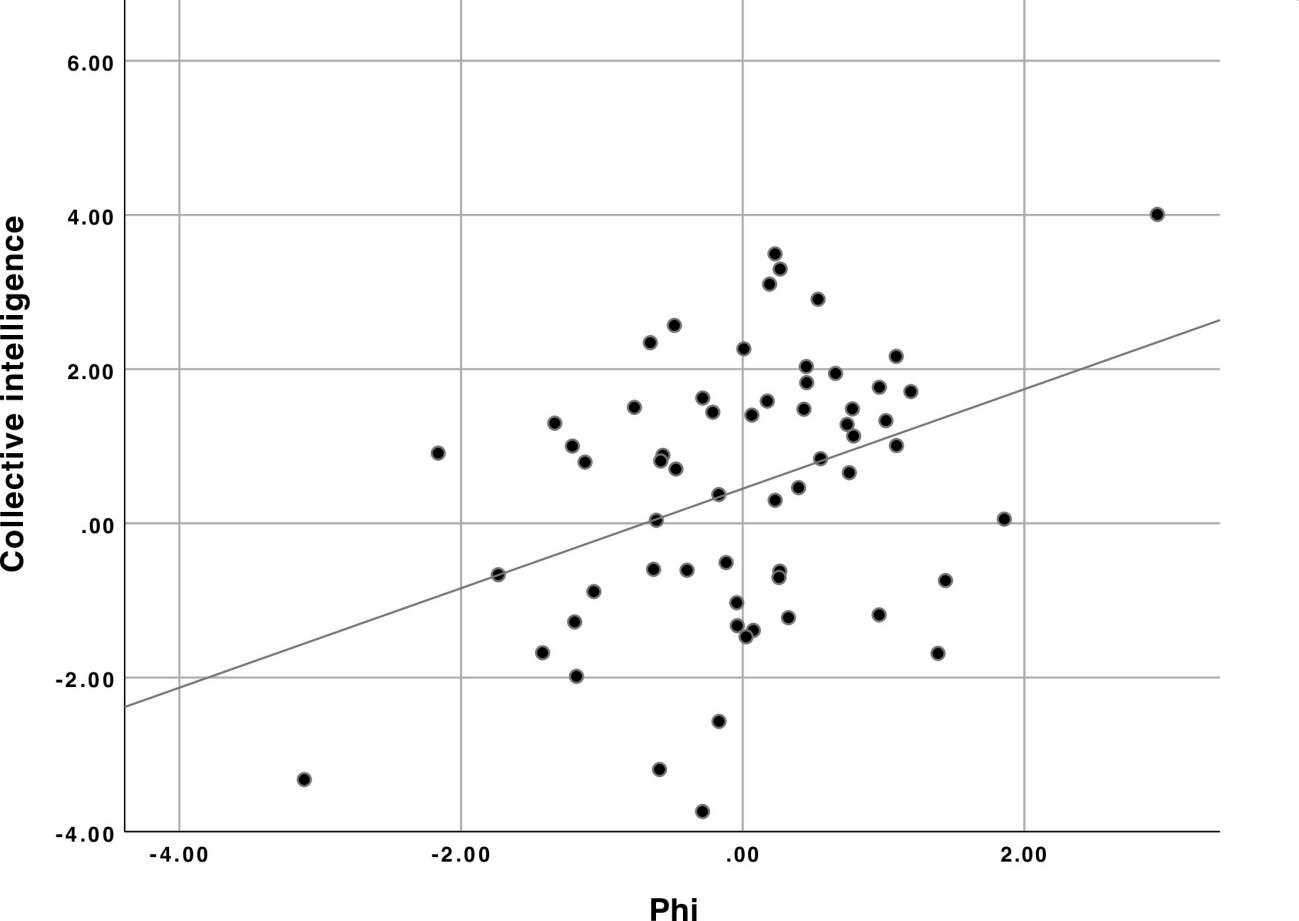
Relationship between phi and collective intelligence. Values shown for both variables are z-scores.

When we calculated phi, it was significantly correlated with the measured collective intelligence of the groups (r = 0.370, p = 0.003; see Methods, Fig 2).

### Study 2: Groups of Wikipedia editors

In Study 2, we analyzed the edit history of Wikipedia articles classified by the Wikipedia community into the following classes, in order of decreasing quality: FA (Featured Article), A, GA (Good Article), B and C [37]. All editors who edited an article were considered members of the “group” for that article, and they were considered “active” when they made an edit.

**Fig 3:**
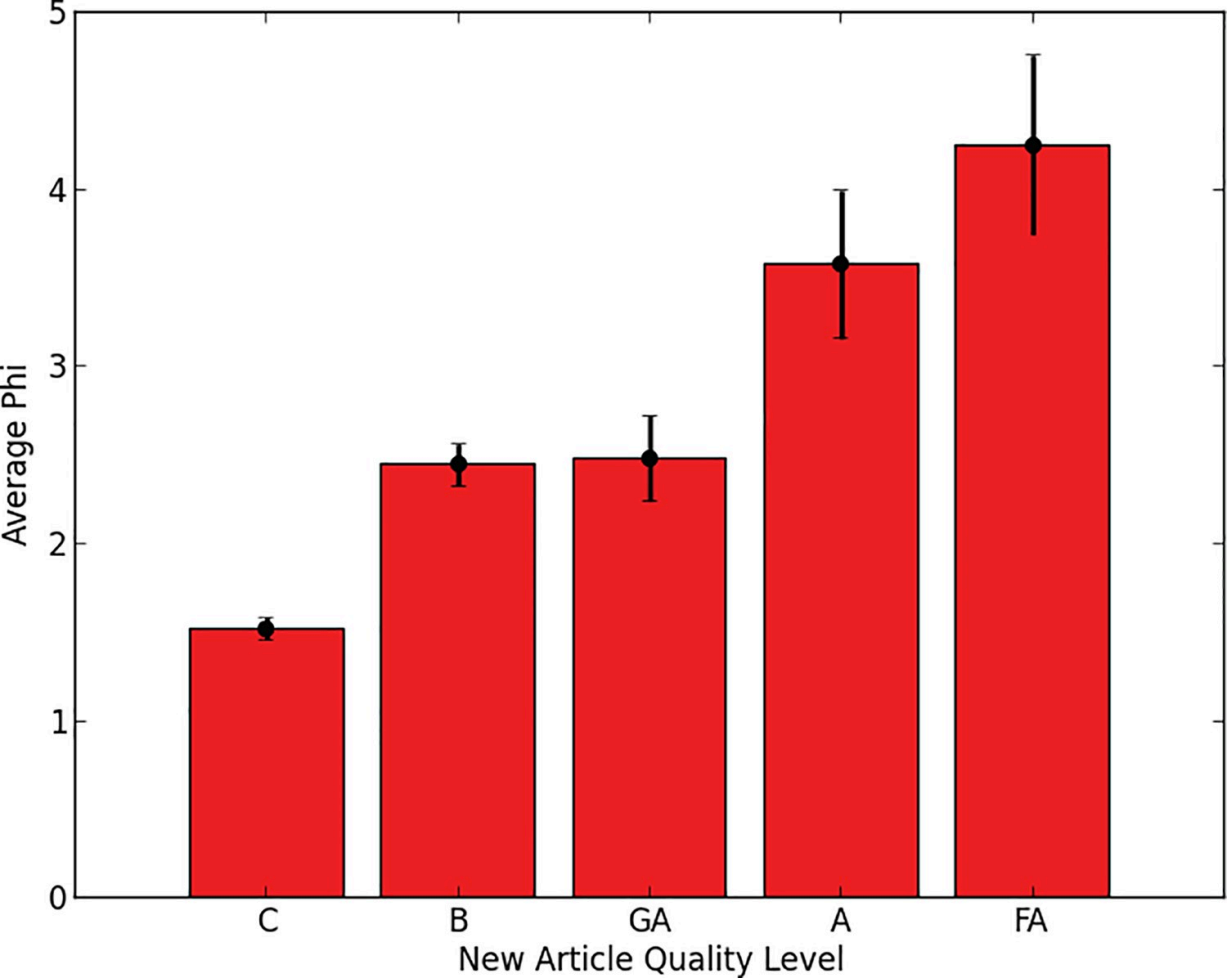
Average phi for groups editing Wikipedia articles of different quality levels in the 60-day period before the articles were promoted to their current quality level. Quality levels are arranged in order of increasing quality. Error bars show standard error.

We found that, in general, groups of editors who produce higher quality articles also have higher phi (Fig 3 and S1 Fig). To test the significance of this effect, we created a regression model that predicted phi for each article from the number of editors, the number of edits per editor and four variables encoding the quality of the article (see S6 Table). Then we assessed the effect of the newly attained quality level in the presence of covariates by a likelihood ratio test between the model without quality as a variable and the full model. This showed that the article quality was significantly correlated with phi, when controlling for the other factors (F = 3.6847, p = 0.0053).

More specifically, pairwise Wilcoxon ranksum tests show that the groups editing FA and A articles have significantly higher phi values than GA and B articles which, in turn, are significantly higher than C (Wilcoxon z-Statistics = 5.6024 p < 0.00001 between C and B and z-statistic = 3.5132, p = 0.0004 between GA and A).

### Study 3: Groups of computers and people on the internet

**Fig 4:**
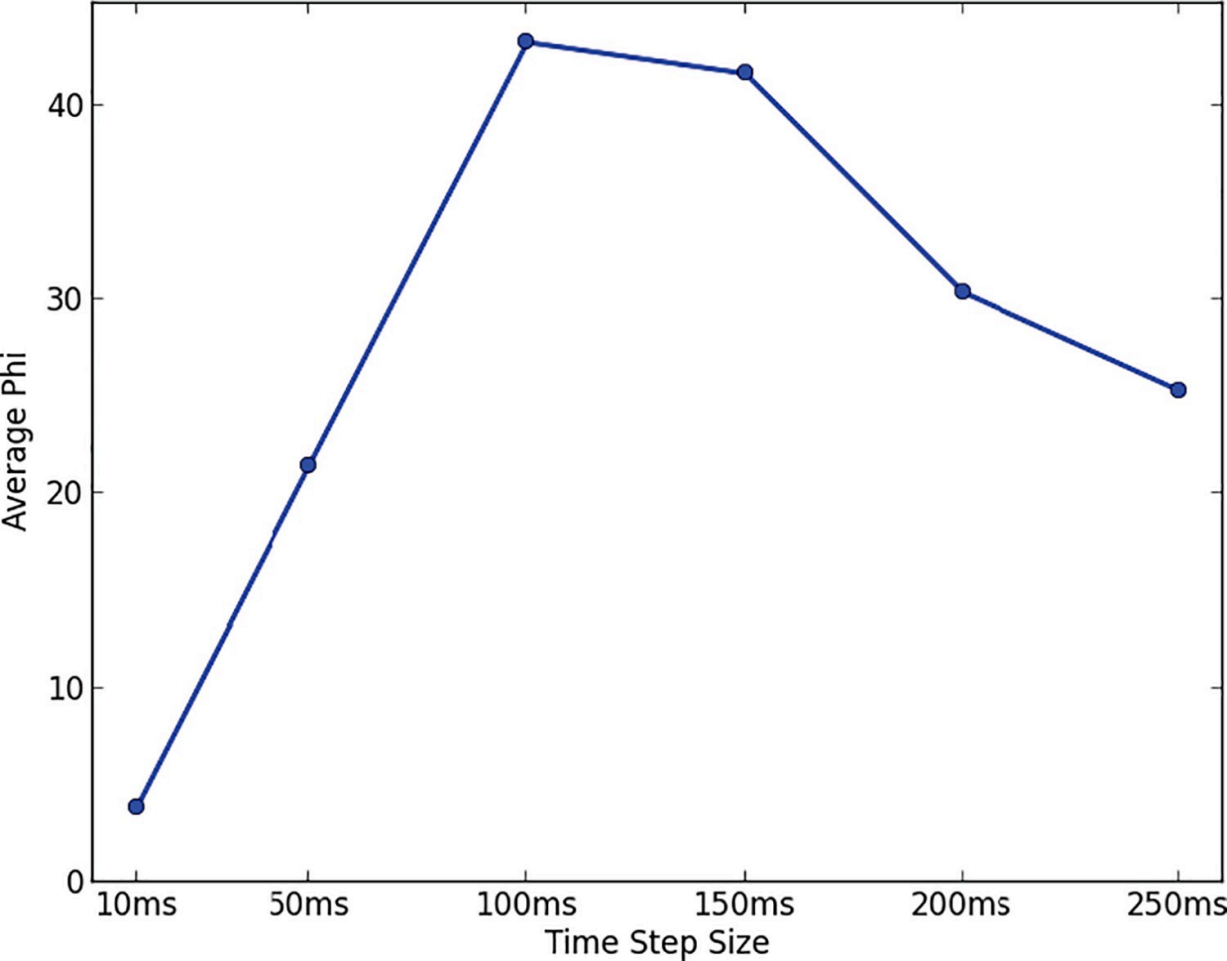
Average phi for Internet traffic computed with different time step sizes.

In Study 3, we applied the phi metric to a sample of the Internet traffic that passed through one Internet backbone over a six-year period [38] (see Methods). We encoded the state of the system in terms of whether a given machine was active (i.e., sent a data packet) at a given time. We picked a time delay of 1 time step and chose the time step size that maximized phi averaged over all years in the dataset (see Fig 4, Methods). In this case, the maximum is at 100 ms which is reassuring since it coincides well with average response times observed over the Internet (see Methods).

**Fig 5:**
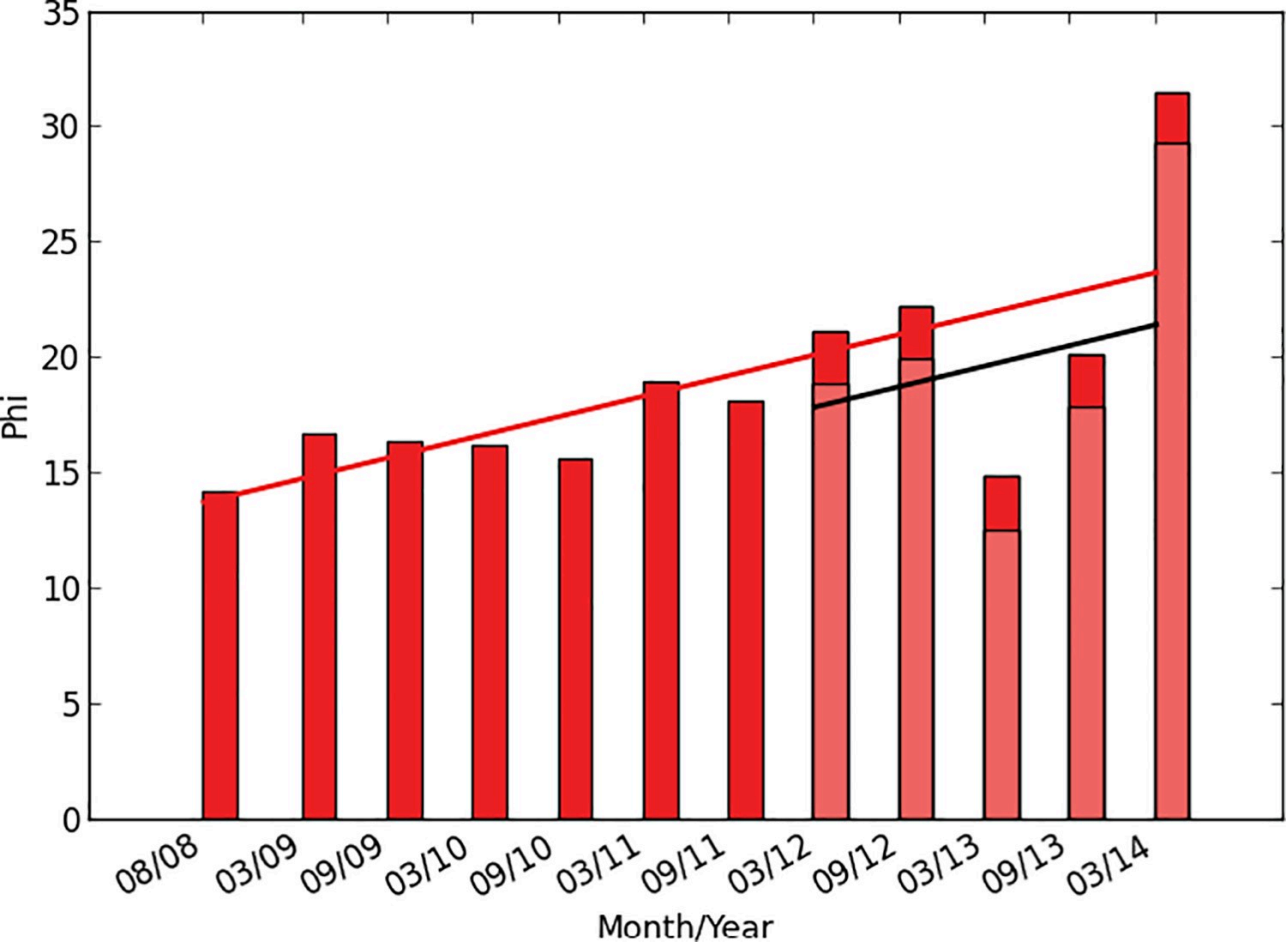
Average phi computed on Internet traffic data over a span of 6 years. Node sampling = random walk, node sample size = 100, time step size = 100 ms. A change in hardware at the recording site between 2011 and 2012 caused a drop in subsequent recorded traffic [39]. The actual traffic in subsequent years is indicated by a horizontal black line and light red bars. The red bars and the red line show values adjusted to compensate for this change. (See details in Methods).

When computing phi, there appears to be a steady upward trend over time. For example, Fig 5 shows one example of a highly significant relationship between the date and phi (β = 1.779, p<0.0001). Similar results were obtained for numerous other sampling methods and parameters (see Methods, S2–S4 Figs). It is important to note that the results do not arise simply from an increasing number of machines in the Internet over time, since the number of machines in the samples analyzed is constant in each case (see Methods).

## Discussion

Together, these results suggest that the concept of integrated information, as formalized by the specific phi metric [23], can be usefully applied to group interactions. To begin with, the time delays at which this measure is maximized are intuitively plausible for a measure of interaction: 2 seconds for face-to-face human groups and 100 ms for machines on the Internet.

### Predicting group performance

More importantly, phi is correlated with various measures of group performance. In 4-person work groups, it is correlated with the groups’ collective intelligence. Previous work has shown that collective intelligence, in turn, predicts a group’s performance on a wide range of other tasks [35,36,40]. Furthermore, in groups of Wikipedia editors, phi is correlated with the quality of the articles the groups edited.

Since phi can be calculated from a relatively small sample of group interactions, this suggests that it might be possible to predict many kinds of group performance, long before a group’s output is complete, merely by measuring phi. This possible use of phi seems plausible because we could interpret phi as a measure of *group collaboration*, and it seems likely that the degree of collaboration in a group could be a good predictor of the group’s performance in many situations.

This use of phi would be analogous to the use of intelligence tests for individuals [41] or groups [36] to predict performance on future tasks. But these intelligence tests are *interventional* measures; they require people to do specific testing activities they would not otherwise have done in order to predict their performance on another task. The versions of phi used here [23], on the other hand, are *observational* measures; as we have seen, they can be calculated merely by observing what people are doing anyway. In this sense, then, phi could provide a relatively easy way of measuring how well a group is working together and using that to predict how well the group will perform on other tasks in the future.

Of course, it is certainly possible that other metrics would have predictive power similar to that of phi and be simpler to compute. Therefore, we believe an important task for future research is to investigate the predictive power of various other metrics. For instance, it is possible that some of the information theoretic or correlational quantities used to compute phi would, themselves, predict performance as well as phi does. Or, perhaps, other measures of complexity (e.g., [42–45]) would be better predictors. And it will certainly be important to compare the predictive power of phi (or its components) with other potential explanatory variables such as (a) the relative participation of different group members [36], (b) the amount of effort and ability members devote to the group’s tasks [46] and (c) different measures of the network topology of the group’s interactions [4].

### Measuring the complexity of group interaction

Unlike the groups in Studies 1 and 2, we don’t have a clear performance measure for the Internet as a whole. However, it is interesting to observe that the same measure of integrated information that predicted performance in the first two studies is increasing over time in the Internet. In other words, we see an increase over time in the particular kind of interaction complexity that phi measures—involving both differentiation and integration among parts.

More generally, it is very intriguing to observe that the same kinds of informational complexity that neuroscientists have postulated are necessary (but not sufficient) for consciousness also appear to be present in well-performing groups.

## Conclusion

In this paper, we have seen how the mathematical concept of integrated information formalizes observations about the importance of differentiation and integration that have arisen, more or less independently, in a number of different disciplines. We have also seen how applying this metric to empirically analyze group interactions can lead to potentially useful predictions of group performance and measurements of interaction complexity.

Much work remains to be done, but, perhaps, applying the concept of integrated information to large groups will be especially useful in understanding the complex kinds of hybrid human-computer systems that are becoming increasingly important in our modern world.

## Methods

The research was approved by Massachusetts Institute of Technology’s Institutional Review Board (IRB). Information about subject recruitment for Study 1 is given below. Studies 2 and 3 involved the analysis of publicly available data which MIT’s IRB confirmed was not subject to their review.

### Study 1: Small work groups

#### Measuring the collective intelligence of small work groups

The data about collective intelligence were collected during a previous study that tested the impact of mode of communication on general group performance [35]. In this study, 68 groups of four people each worked together on a set of diverse online tasks. The tasks included both verbal and nonverbal activities of the following types: generating, choosing, remembering, sensing, and taking physical actions. For instance, tasks included brainstorming uses for a brick, solving Raven’s Matrices problems from a standardized intelligence test, remembering features of complex videos and images, and copying complex text passages into a shared online editor. Detailed task descriptions and descriptive statistics are included in [35], and summary task descriptions are in S1 and S2 Tables. The subjects were recruited via Internet advertisements in the Boston area during 2012, and consent was obtained in writing. Subjects were required to be at least 18 years old and to have vision that was normal or corrected to normal. The participants ranged in age from 18 to over 65.

All group members used individual laptop computers to work on the shared online tasks. In one condition, the group members were seated near each other and were able to communicate face-to-face while solving the tasks. In the other condition, the group members were seated far apart and were only able to communicate via the text chat functionality built into the online system.

To determine the collective intelligence scores for the different groups, we performed a factor analysis of the groups’ scores on the different tasks. As with previous work [36], the first factor in these analyses explained around 40% of the variance in the groups’ performance on all the tasks. We treated each group’s score on this first factor as the group’s collective intelligence. This collective intelligence score, therefore, is a weighted average of the group’s scores on all the tasks with the weights chosen to maximize the predictive power for performance on all the tasks. In this sense, the collective intelligence score for a group is exactly analogous to individual intelligence test scores for individuals [36,41].

#### Calculating phi for small work groups

S3 Table summarizes the methods used in all three studies. For analyzing the data from Study 1, we used the phi metric that Barrett and Seth [23] call Φ_E_ (“empirical phi”). This metric is based upon the theoretical definition of phi by Balduzzi and Tononi [13] and assumes that the system being analyzed is stationary. It can be written as:

$$\Phi_{E}[X;\tau ]=I(X_{t-\tau };X_{t})-\sum_{k=1}^{2}I\;(M_{t-\tau }^{k};M_{t}^{k})$$ (2)

where ***X*** is a stochastic system, τ is the time delay with respect to which phi is measured, ***X***_*t*_ is the state of the system at time *t*, and M^1^ and M^2^ are subsets of ***X*** chosen such that they constitute a minimum information bipartition (MIB) of ***X*** (see [23] for details of how to obtain the MIB).

***I*** (***X*,*Y***) is the mutual information between ***X*** and ***Y*** which is defined as the reduction in uncertainty (entropy), about X, knowing the outcome of Y:

I(X;Y)=H(X)–H(X|Y). (3)

Thus Φ_E_ is another way of calculating the information generated by the system as a whole that is more than just the sum of its parts.

To use this metric, we recorded communication in different ways for the two conditions. For the face-to-face condition, each group member had an individual microphone. This resulted in four time-aligned audio tracks. We first used software to split the audio tracks into time steps of 200 ms each. The software then determined for each time step, who, if anyone, was speaking. To do this, the software analyzed which group members’ audio volumes were above a threshold level. This level was optimized based on “ground truth” data obtained from human observer ratings of who was speaking for a limited subset of the data. The next step suppressed the audio tracks that picked up muted versions of someone else’s speech. The final step merged speaking turns of a single speaker that were 400ms or less apart (e.g. someone making a brief pause during a speaking turn).

This procedure thus yielded, for each team, a state vector that encoded everyone who was speaking at a specific time step with a 1 and everyone else with a 0. We then applied the phi metric to this state vector.

For the online condition, we used software to analyze the chat transcripts. We encoded each line of chat as one time step. During this time step, the group member who chatted is encoded as 1 (for active) and all other group members are encoded as 0 (for inactive). This encoding leads to a situation (unlike with the face-to-face groups) where only one person can be active at any given time. We then computed the phi metric on this state dataset.

We also needed to determine the time delay with respect to which phi would be calculated. For the online condition, we expect to see an influence of what is said in one comment on the next comment, so we set the time delay to one “timestep,” that is, the time from one textual comment to the next one.

For the face-to-face groups, we don’t expect the actions of one group member to immediately influence the actions of another one. Instead, we would expect time delays on the order of a few seconds, the approximate time it takes for a person to hear and respond to what someone else says. To determine the exact time delay, we plotted average phi for different time delays. As described in the main text and shown in Fig 1, there was a clear peak at around 2 seconds, so we used this value.

Since phi was computed in very different ways in the two conditions, we normalized the phi scores by condition. Then when we performed a hierarchical regression analysis of the effects on collective intelligence of phi and condition (face-to-face vs. online), we found a significant effect of phi alone but no significant effects of adding condition or the interaction between phi and condition (S4 Table). We also obtained similar results when the collective intelligence scores were normalized by condition. Therefore, we pooled data from the two conditions and found an overall correlation of r = 0.370 (p = .003) between collective intelligence and phi (see scatterplot in Fig 2).

We also investigated the two potential outliers shown in the upper right and lower left corners of the scatterplot. Using the outlier labeling method with the value of k = 2.2 recommended by [47], we found that the upper right point is not an outlier and the lower left point is on the border. The lower left point would be considered an outlier for a value of k = 2.2, but not for a value of k = 2.23 which is within the rounding error of the recommended value, and the point would definitely not be an outlier with the value of k = 3.0 which is sometimes used [47]. Even if the lower left point is eliminated from the analysis, the correlation of phi and collective intelligence is still significant (r = .290, p = .025).

Note that, in this study and the other two, we assume, but do not test for, stationarity of the time series of state vectors that are used to calculate phi. We believe that the results reported above about correlations between phi and other variables are of interest, in any case, even if they are caused, in part, by factors that led to non-stationarity in the systems. However, as noted in the Discussion above, we also believe that an important focus for future work would be to examine many alternative factors that might explain our results, including any that might have involved non-stationarity in the systems.

### Study 2: Groups of Wikipedia editors

#### Measuring quality of Wikipedia articles

In this study, we analyzed the edit histories of the articles in Wikipedia’s Vital Articles list [48]. At the time of downloading, this included 1000 articles spanning a wide range of rated quality levels, topics, and popularity. We discarded the Wikipedia front-page article since it had an order of magnitude more edits than any other article in the list and thus was as a clear outlier. This left 999 articles that we analyzed.

From the edit histories of these articles, we parsed the quality level of the articles for each edit step giving us the points in time when changes in quality occurred. We then analyzed the 30-, 60-, and 90-day periods before each quality change, discarding the entire article for periods in which only one or two editors were active (see descriptive statistics in S5 Table).

#### Calculating phi for groups of Wikipedia editors

We computed phi for each article in the 30-, 60-, and 90-day periods before each quality change. As with the chat transcripts in Study 1, we encoded each edit as a single time step. An editor was considered to be active if he or she edited the Wikipedia article in question at that time step and inactive otherwise.

However, we could not compute phi for Study 2 using the Φ_E_ metric used in Study 1 for two reasons. First, as the number of nodes in the network grows, it becomes increasingly difficult to obtain enough data to accurately estimate all the relevant entropies using Φ_E_ [23]. To deal with this problem, we used the phi metric that Barrett and Seth call Φ_AR_ (“auto-regressive phi”). This metric provides reasonable estimates for both Gaussian and non-Gaussian systems with smaller amounts of data [23] and can be written as:

$$\varphi_{AR}[X;\tau ,\{M^{1},M^{2}\}]=\frac{1}{2}log\left\{\frac{det\sum \left(X\right)}{det\sum \left(E^{X}\right)}\right\}-\sum_{k=1}^{2}\frac{1}{2}log\left\{\frac{det\sum \left(M^{k}\right)}{det\sum \left(E^{M^{k}}\right)}\right\}$$ (4)

where M^1^ and M^2^ are a bipartition of the data, detΣ(X) is the determinant of the covariance matrix of X, and E^Mk^ and E^X^ are residuals in regression equations that estimate states of the system at one time based on knowledge of the system state at another time. To compute this version of phi, we used a MATLAB toolbox provided by Adam Barrett [23].

The second problem that arises with large systems is that determining the minimum information bipartition (MIB) requires enumerating all possible bipartitions of the dataset. Since the number of these bipartitions grows exponentially with the size of the network, this method quickly becomes computationally infeasible. To avoid these problems, we used “atomic” partitions in determining phi as recommended by [31,33]. With this approach, each node is considered as its own partition M^k^ and the summation in the second term is done over all these.

We verified the validity of this atomic measure on our dataset by computing the normal phi and the atomic phi for all edit histories from the Wikipedia dataset with 14 editors or less, 14 being the largest number where enumerating all bipartitions is still computationally feasible for all articles. The values of the atomic phi are higher but the correlation between the original phi and the atomic phi was highly significant (r = 0.83, p<0.001).

In some cases in our data, the Φ_AR_ algorithm became numerically unstable and was unable to return a value at all. In other cases, the algorithm returned extreme values (below 0 or greater than the number of nodes) that would have been theoretically impossible in the original definitions of phi, which Φ_AR_ is intended to estimate. Specifically, Φ_AR_ is known to sometimes produce values less than 0, even though this cannot happen in the original definition of phi [24, 25]. And for binary values like those we used, atomic phi cannot be greater than the number of nodes (since, in the original definition of atomic phi, the conditional entropy added by each node cannot be greater than 1 bit). These problems usually occurred in cases where many nodes had (little or) no variance in their activities (e.g., the nodes were almost always on or almost always off). In these cases, the state vector matrices often became rank deficient, and the algorithm was unable to compute a valid value for phi.

Since nodes with (little or) no variance have (little or) no entropy, they also have (little or) no effect on phi. Therefore, in cases where the algorithm returned no value (or an extreme value) for phi, we simply dropped the 5% of nodes with the least variance and reran the computation, repeating this procedure until the algorithm did return a valid, non-extreme value. In Study 2, these problems occurred in 19.5% of the cases, and when they occurred, we had to repeat the procedure 2.27 times on average. In other words, we corrected for numerical instabilities and extreme values in the calculation of phi by removing a small number of low-variance nodes that would have had little effect on phi in any case. This insured that all the data analyzed was for groups of nodes for which valid, non-extreme values of phi could be computed.

### Study 3: Groups of computers and people on the internet

To analyze Internet traffic, we used a database compiled by the Cooperative Association for Internet Data Analysis (CAIDA) [38]. This database includes records of the data logged by two high-speed monitors on a commercial backbone link on the Internet. The monitors are in Chicago and San Jose, and we chose the one in San Jose since it provides a longer undisrupted history (from 2008 to the present). We analyzed datasets separated by approximately 6-month intervals during the period (usually every March and September).

Since the volume of Internet backbone traffic is huge, the database includes only one hour of data for each month, and we limited our analysis to one minute of this data for the months we analyzed. We picked the fourth minute of each hour to avoid any unusual activities in the first minute of the hour (such as special programs that operate automatically at the beginning of each hour).

The database contains a trace of each packet of information sent, including an anonymized version of the Internet Protocol (IP) address for the origin and destination node of each packet. Each node (or “host”) is a different computer, such as an end user’s laptop, a mail server, or a web server for Google, Amazon, and other web service providers. The IP addresses for these nodes are anonymized in such a way that each real IP address always matches to the same anonymized counterpart.

Descriptive statistics for the dataset, after unpacking and parsing (including, for example, removing IPv6 and unreadable packets) are shown in S7 Table.

#### Calculating phi

We calculated phi for Study 3 using the same phi metric used for Study 2. To do this, we characterized the state of the system in terms of which nodes were active (in the sense of sending an information packet) at a given time. We also determined a time delay with respect to which to calculate phi. In order to do these things, several other steps were also needed.

#### Sampling nodes

As shown in S7 Table, the number of nodes sending packets in the months we analyzed ranged from about 200,000 to 1.6 million. We know of no method for calculating phi that is computationally feasible and numerically stable for systems with anything remotely approaching this number of nodes, so, before calculating phi, we needed to subsample the nodes to be analyzed. Ideally, these sampling methods should select subsets of nodes whose activity relationships are representative of those in the whole sample. Therefore, the first two methods we used were the two methods for sampling from large graphs that were found by Leskovec and Faloutsos [47] to best retain the network properties of the graphs.

In describing these methods, we denote by **S** the set of all nodes in a sample of information packets, and by **A** ⊂ **S** the subsample of nodes to be analyzed. Bold lower case letters indicate single nodes. D(**a**) refers to the set of all destination nodes to which node **a** sent a packet in the sampled period and D(**A**) is the set of all destinations for any node in **A**. We do not allow duplicate nodes in **A**.

The two methods we used were:

- *Random walk*. We first pick a random node **x ∈ S**, add it to **A,** and make it the active node. We then randomly pick a new active node **y ∈** D(**x**) and add it to **A**. At each step, we continue by doing one of two things. With probability 0.85, we pick a new active node from the destinations of the current active node. And with probability 0.15, we return to **x** and start a new path from there. If we run out of new nodes to visit (e.g. in the case of a small isolated subset) we pick a new starting node **x**. This method is repeated until we have reached the sampling goal. As stated by [47] the return probability of 0.15 is the standard value picked in literature.
- *Forest fire*. We first pick a random starting node **x ∈ S**, add it to **A,** and make it the active node. Next we pick a random number *n* from a geometric distribution with mean 2.3 (the value suggested by [47]), randomly pick *n* nodes from D(**x),** and add these nodes to **A**. The procedure continues by selecting new active nodes from **A** and repeating the process until the required number of nodes is reached. If at any point, there are no nodes left in D(**A**) that are not already in **A,** then a new random starting node **x ∈ S** is selected and added to **A**.
  For comparison, we also used two other simple sampling methods:
- *Breadth first*. We randomly pick **x ∈ S** as our starting node and add it to **A**. We then iteratively add to **A** all nodes to which nodes in **A** sent packets (i.e. D(**A**)) until we reach our sampling goal. If there are no more nodes in D(**A**) that are not already in **A,** we pick a new starting node **x ∈ S** and continue from there.
- *Random nodes*. We randomly pick **x ∈ S** and add it to **A** until we reach our sampling goal. Note that this method selects a small number of nodes (e.g., 100) completely randomly from a much larger set (e.g., several hundred thousand nodes). Therefore, even if there are substantial interactions among nodes of the type phi measures, this node sampling method may not detect them very well. However, we still include it for comparison purposes.

For each date and each node sampling method, we created 100 different random subsamples of nodes. We then computed phi on the resulting state vectors and averaged the results across all 100 different random subsamples.

#### Determining time step size and time delay

To characterize the state of the system, we needed to determine the size δ of the time steps into which activity data will be grouped (i.e. for which we assume all the data packets are sent at the same time). We also needed to pick a time delay τ with respect to which phi will be calculated. These two factors depend on each other logically. For instance, if there are true interactions at a timescale of 100 ms, we could detect them with phi by, for example, setting δ = 100 ms and τ = 1 time step or by setting δ = 50 ms and τ = 2 time steps.

To make the search space of possibilities more manageable, we fixed the time delay τ = 1 and selected the time step size δ that maximized phi when averaged across all the dates in our analysis. In calculating phi for this purpose, we made the following assumptions: (a) node sampling was done using the random walk method, and (b) the other corrections described below were made. This resulted in a time step size δ of 100 ms (see Fig 4). We also obtained similar results for other combinations of parameters.

This corresponds very well with typical response times observed on the Internet. As noted by [49], the typical “round trip” time for data on the Internet to travel from point A to point B and back is about 200 ms. If we make the reasonable assumption that the processing time on the remote machine is minimal, then the delay is almost entirely due to time spent travelling back and forth on the network, so each one-way trip would be about 100 ms. The time delay relevant for calculating phi is the delay for one-way travel plus the time for the remote machine to respond, so these numbers correspond very well.

#### Determining node sample size

Based on preliminary experiments with our data, we found that the computations for phi often became numerically unstable and very computationally expensive at around 200 nodes. To avoid these problems we picked a standard node sample size of 100 nodes. As noted below, however, the results were also similar with samples of 150 and 200 nodes.

#### Correcting for numerical instabilities

We used the same method to correct for numerical instabilities as used in Study 2. In Study 3, invalid values occurred initially in 67.14% of the cases, but they disappeared after repeating an average of 2.12 times the procedure of dropping low variance nodes.

#### Correcting for hardware change at the recording site

The hardware at the recording site was upgraded in the time period between September 2011 and March 2012, which led to a noticeable drop in the phi values. To correct for this, we added an indicator variable to our linear model that indicates if the date is before or after March 2012. This allowed us to extrapolate the corrected phi according to the model. For readability reasons, the graphs in Fig 5 and S2–S4 Figs show the extrapolated values in red, the uncorrected values in light red, the regression line for the corrected values as a red line and the regression line for the uncorrected values as a black line.

#### Results

Using the procedures just described, we calculated the value of phi over time for four node sampling methods (random walk, forest fire, breadth first, and random nodes). The resulting graphs are shown in Fig 5 and S2 Fig. In all cases except random node sampling, the relation between phi and year is positive and very significant (see Table 1). As noted above, we did not expect the random node sampling method to be very effective at detecting interactions of the sort phi measures, so it is not surprising that the results were not significant in this case.

**Table 1:** Regression coefficients for predicting phi from date with four different node sampling methods.

	Node sampling method			
	Random Walk	Forest Fire	Breadth First	Random Nodes
Regression coefficient	1.675***	1.676***	1.715***	-0.26

*** = p < 10^−8^

#### Robustness check for time step size

As noted above, the main results were calculated with a time step size δ = 100 ms which maximized the value of phi. However, S3 Fig shows that using time step sizes of 50 ms or 150 ms also yields similar results.

#### Robustness check for node sample size

As noted above, the main results were calculated with a node sample size of 100 nodes. However, S4 Fig shows that using sample sizes of 150 or 200 also yield similar results.

#### Robustness check for number of packets sampled

As shown in S7 Table, the number of packets sent in the minutes we studied is not constant over the dates we studied. To investigate whether the variable number of packets could have affected the results, we also investigated a different method for sampling packets. With this alternate method, we analyzed only the first 10,000,000 packets in each minute, since this is the maximum (round) number of packets present for all dates. S8 Table shows the regression coefficients for this sampling method. We see again that date is a significant predictor of phi in this case for all four sampling methods.

## Supporting information

S1 Fig Average phi for groups editing Wikipedia articles of different quality levels in the 30-day period (A) and the 90-day period (B) before the articles were promoted to their current quality level.
(DOCX)

S2 Fig **Average phi over time for various node sampling methods (top left to bottom right: Random Walk, Forest Fire, Breadth First and Random Nodes)**. In all cases node sample size = 100, and time step size δ = 100 ms.
(DOCX)

S3 Fig Average phi plotted over time (node sampling = random walk, node sample size = 100, time step size δ = 50 ms (left) and 150ms (right). For 50ms, β = 0.8227, p = 0.000007; for 150ms, β = 1.333, p = 0.002.
(DOCX)

S4 Fig **Average phi plotted over time (node sampling = random walk, node sample size = 150 (left) and 200 (right), time step size δ = 100 ms)**. For 150 nodes, β = 0.4477, p = 0.018; for 200 nodes, β = 0.6535, p = 0.00026.
(DOCX)

S1 Table Task categories and verbal vs. non-verbal dimensions in the Collective Intelligence task battery (reproduced from [23]). (DOCX)

S2 Table Description of tasks used to measure collective intelligence of groups. (DOCX)

S3 Table Summary of methods used to calculate phi in different studies. (DOCX)

S4 Table Hierarchical regression results for predicting collective intelligence from phi, condition (face-to-face vs. online), and the interaction of phi and condition (n = 61 groups). (DOCX)

S5 Table **Descriptive statistics of the number of articles, editors, edits, and edits per editor in various periods before quality changes in the Wikipedia dataset** (time windows of 30-, 60-, and 90-days shown in panels A, B, and C, respectively).
(DOCX)

S6 Table Regression results when predicting phi for each article from number of edits, average number of edits per editor, and newly acquired quality level of the article. (DOCX)

S7 Table Number of information packets, origin nodes and destination nodes for each month analyzed. (DOCX)

S8 Table Regression coefficients for predicting phi from date with four different node sampling methods while only looking at a fixed number of packets (10,000,000 packets). (DOCX)
