# Supplementary material for: Integrated information as a metric for group interaction
Source: PLoS One. 2018 Oct 11;13(10):e0205335. doi: 10.1371/journal.pone.0205335 (PMC6181355; doi:10.1371/journal.pone.0205335)
Supplement: S1 Fig — Average phi for groups editing Wikipedia articles of different quality levels in the 30-day period (A) and the 90-day period (B) before the articles were promoted to their current quality level. (DOCX) [file pone.0205335.s001.docx]

| 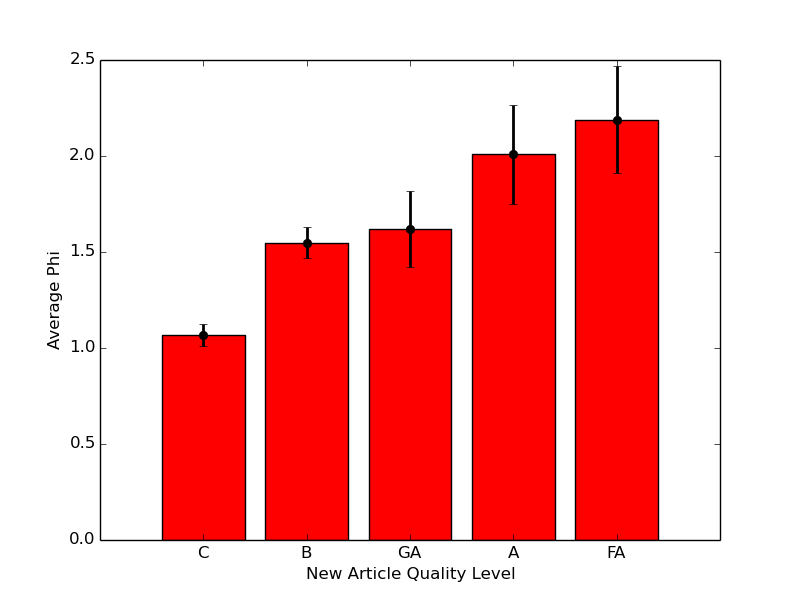  A | 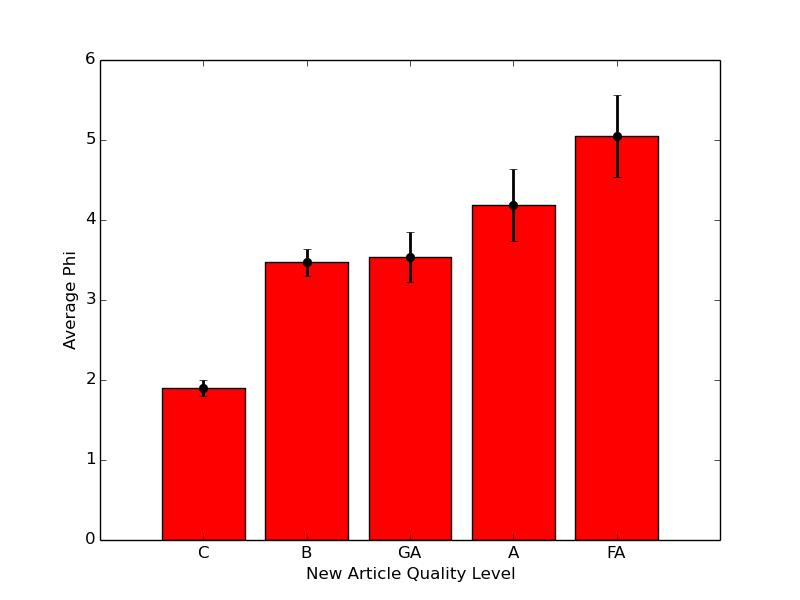  B |
| --- | --- |

**S1 Fig.** **Average phi for groups editing Wikipedia articles of different quality levels in the 30-day period (A) and the 90-day period (B) before the articles were promoted to their current quality level.**
