# Supplementary material for: Integrated information as a metric for group interaction
Source: PLoS One. 2018 Oct 11;13(10):e0205335. doi: 10.1371/journal.pone.0205335 (PMC6181355; doi:10.1371/journal.pone.0205335)
Supplement: S2 Fig — Average phi over time for various node sampling methods (top left to bottom right: Random Walk, Forest Fire, Breadth First and Random Nodes). In all cases node sample size = 100, and time step size δ = 100 ms. (DOCX) [file pone.0205335.s002.docx]

| 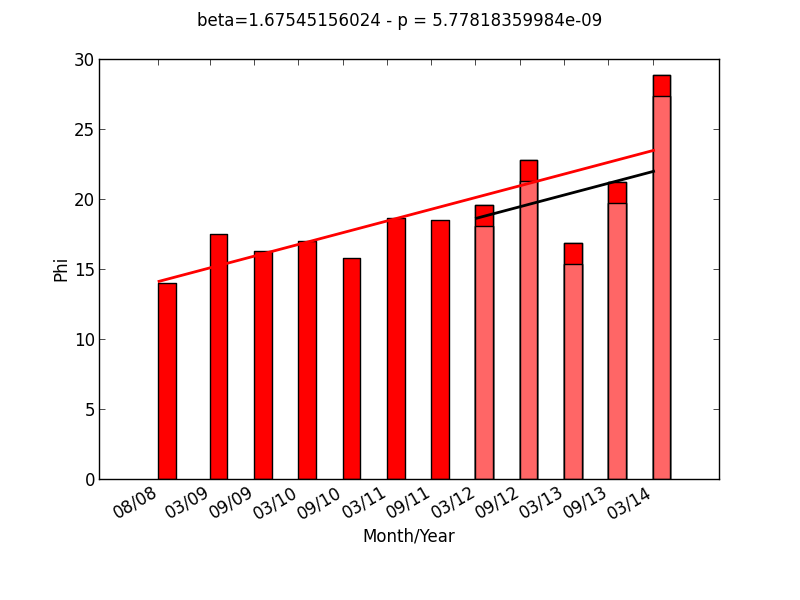 | 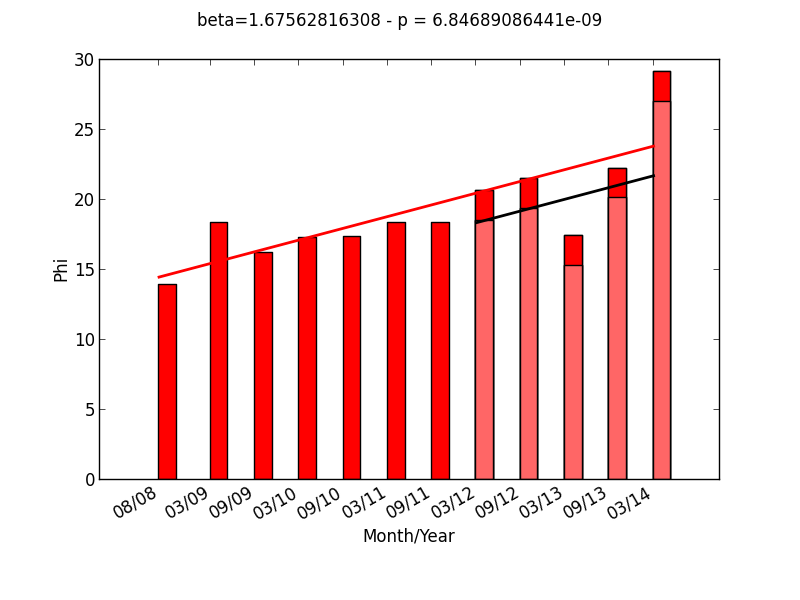 |
| --- | --- |
| 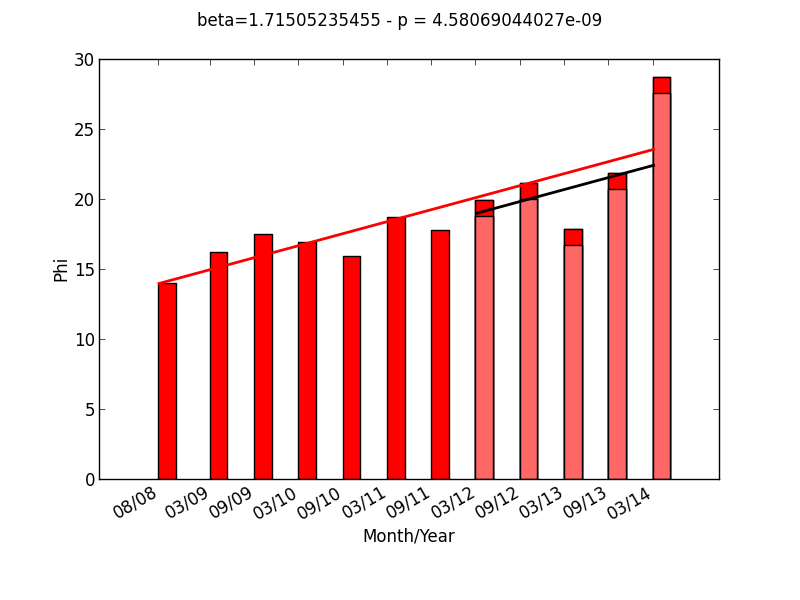 | 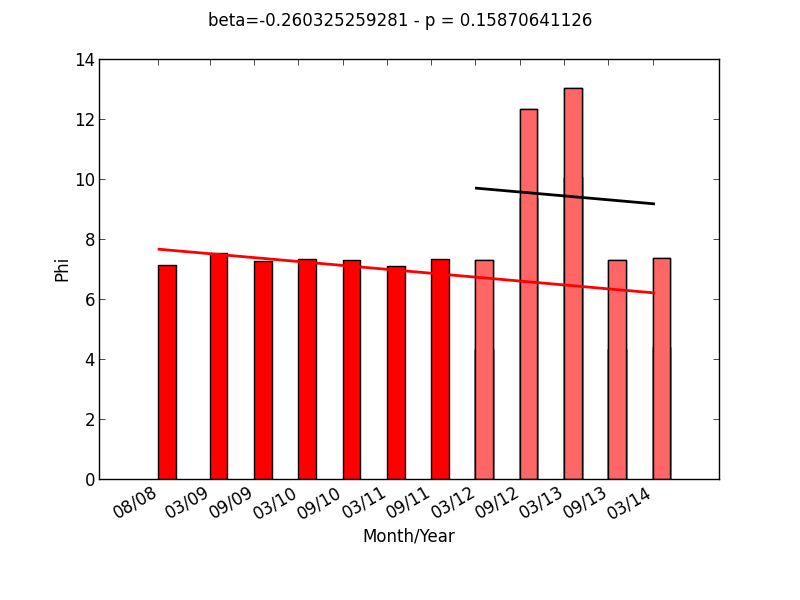 |

**S2 Fig**: **Average phi over time for various node sampling methods (top left to bottom right: Random Walk, Forest Fire, Breadth First and Random Nodes).** In all cases node sample size = 100, and time step size δ = 100 ms.
