# Supplementary material for: Integrated information as a metric for group interaction
Source: PLoS One. 2018 Oct 11;13(10):e0205335. doi: 10.1371/journal.pone.0205335 (PMC6181355; doi:10.1371/journal.pone.0205335)
Supplement: S3 Fig — For 50ms, β = 0.8227, p = 0.000007; for 150ms, β = 1.333, p = 0.002. (DOCX) [file pone.0205335.s003.docx]

| 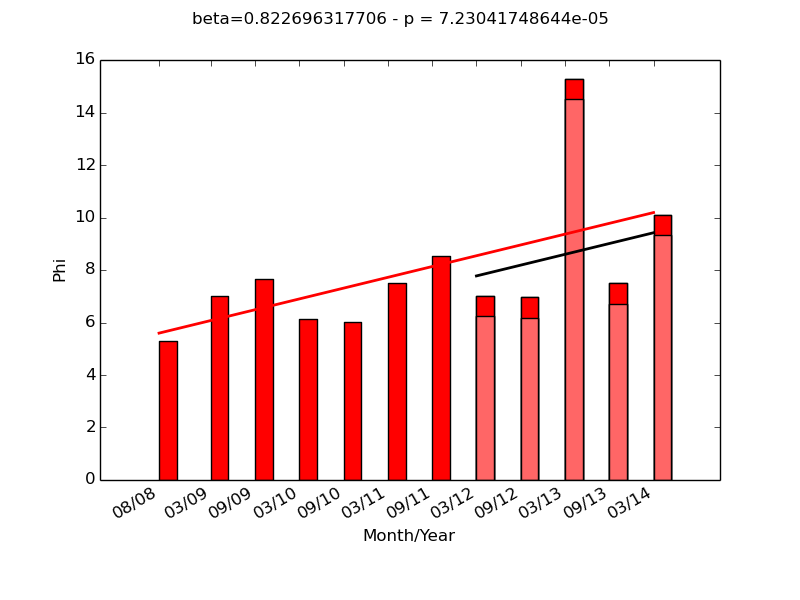 | 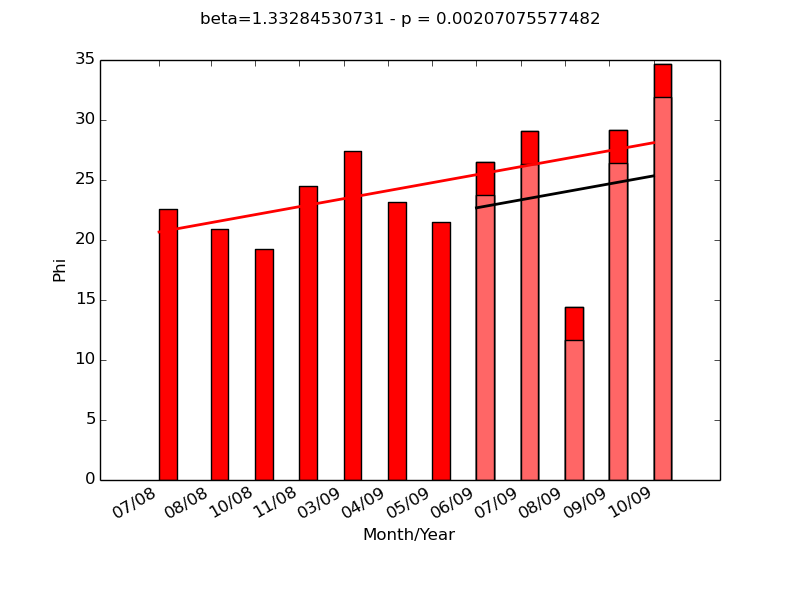 |
| --- | --- |

**S3 Fig**: **Average phi plotted over time (node sampling = random walk, node sample size = 100, time step size δ = 50 ms (left) and 150ms (right).** For 50ms, β = 0.8227, p = 0.000007; for 150ms, β = 1.333, p=0.002.
