# Supplementary material for: Integrated information as a metric for group interaction
Source: PLoS One. 2018 Oct 11;13(10):e0205335. doi: 10.1371/journal.pone.0205335 (PMC6181355; doi:10.1371/journal.pone.0205335)
Supplement: S4 Fig — Average phi plotted over time (node sampling = random walk, node sample size = 150 (left) and 200 (right), time step size δ = 100 ms). For 150 nodes, β = 0.4477, p = 0.018; for 200 nodes, β = 0.6535, p = 0.00026. (DOCX) [file pone.0205335.s004.docx]

| 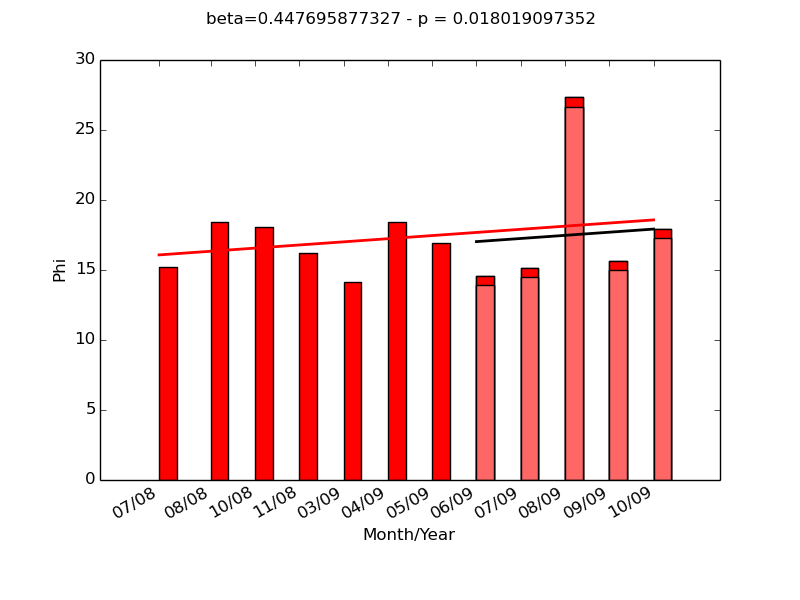 | 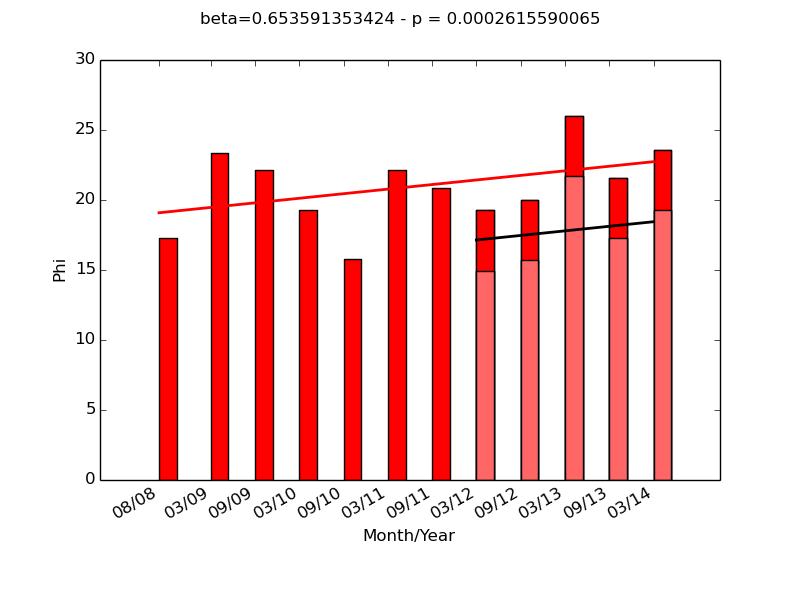 |
| --- | --- |

**S4 Fig**: **Average phi plotted over time (node sampling = random walk, node sample size = 150 (left) and 200 (right), time step size δ = 100 ms).** For 150 nodes, β = 0.4477, p = 0.018; for 200 nodes, β = 0.6535, p=0.00026.
