# Supplementary material for: Integrated information as a metric for group interaction
Source: PLoS One. 2018 Oct 11;13(10):e0205335. doi: 10.1371/journal.pone.0205335 (PMC6181355; doi:10.1371/journal.pone.0205335)
Supplement: S1 Table — (DOCX) [file pone.0205335.s005.docx]

**S1 Table. Task categories and verbal vs. non-verbal dimensions in the Collective Intelligence task battery** (reproduced from (23))

| **Task Category** | **Verbal** | **Non-Verbal** |
| --- | --- | --- |
| 1. Generating | Brainstorming Words | Brainstorming Uses for a Brick  Brainstorming Equations |
| 2. Choosing | Unscramble Words  Judgment Slogans | Matrix Reasoning  Sudoku  Judgment Picture  Judgment Pages |
| 3. Executing | Typing Text | Typing Numbers |
| 4. Remembering | Memory Words* | Memory Video  Memory Images |
| 5. Sensing | Detection Words | Detection Images |

* Due to technical problems with administration of the “Memory Words” task, it was excluded from the analysis.
