# Supplementary material for: Integrated information as a metric for group interaction
Source: PLoS One. 2018 Oct 11;13(10):e0205335. doi: 10.1371/journal.pone.0205335 (PMC6181355; doi:10.1371/journal.pone.0205335)
Supplement: S2 Table — (DOCX) [file pone.0205335.s006.docx]

**S2 Table.** **Description of tasks used to measure collective intelligence of groups.**

| **Task Type** | **Description** |
| --- | --- |
| Brainstorming | Groups had to collectively come up with as many as possible of the following: (a) uses for a brick, (b) words that started with S and ended with N, and (c) equations that equal 10 with certain constraints on operators and values used. Points were given for number of answers and uniqueness of answers. |
| Unscrambling | The subjects were awarded points for correctly identifying words whose letters were randomly scrambled |
| Matrix Reasoning | Solving a set of Raven’s Advanced Progressive Matrices, a standardized test of general fluid intelligence. |
| Sudoku | Solving a Sudoku puzzle. Points were awarded for number of correct answers |
| Judgment | Groups had to predict how a larger population would rate the quality of images and slogans. They also had to estimate the number of pages in a book based on a picture of the book. |
| Typing | The groups had to copy a complex text passage and a complex series of numbers into a shared workspace similar to Google Docs. Scoring was based on number of items copied correctly with significant penalties for incorrect and skipped items. Therefore, it was important for groups to carefully coordinate their work to avoid duplications and long sequences of skipped items. |
| Memory | Groups were shown complex videos, images, and sequences of words and then asked to answer a set of questions about the items they had seen. |
| Detection | Groups answered questions about a grid of small images such as “What is the most frequent object in the grid?” |
