# Supplementary material for: Integrated information as a metric for group interaction
Source: PLoS One. 2018 Oct 11;13(10):e0205335. doi: 10.1371/journal.pone.0205335 (PMC6181355; doi:10.1371/journal.pone.0205335)
Supplement: S3 Table — (DOCX) [file pone.0205335.s007.docx]

**S3 Table:** **Summary of methods used to calculate phi in different studies**

|  | | **Study 1** | | **Study 2** | **Study 3** |
| --- | --- | --- | --- | --- | --- |
|  |  | Face-to-face condition | Online condition |  |  |
| **Definition of phi** | | Φ_E_ | Φ_E_ | Φ_AR_ | Φ_AR_ |
| **Nodes** | | Humans  (in face-to-face group) | Humans  (in online chat) | Wikipedia editors | Computers  (on the Internet) |
| **States** | **1** | Talking | Chatting | Editing | Active  (sending a data packet) |
|  | **0** | Not talking | Not chatting | Not editing | Not active |
| **Time step** | | 200 ms | Each message | Each edit | 100 ms |
| **Time delay** | | 2 s | 1 time step | 1 time step | 100 ms |
| **No. of nodes** | | 4 | 4 | Min: 2 Max: 152 Mean: 15 | Min: 200,000 Max: 1,600,000 Subsampled to 100 |
| **Partition** | | Minimum Information Bipartition | Minimum Information Bipartition | Atomic | Atomic |
