# Supplementary material for: Integrated information as a metric for group interaction
Source: PLoS One. 2018 Oct 11;13(10):e0205335. doi: 10.1371/journal.pone.0205335 (PMC6181355; doi:10.1371/journal.pone.0205335)
Supplement: S4 Table — (DOCX) [file pone.0205335.s008.docx]

**S4 Table:** **Hierarchical regression results for predicting collective intelligence from phi, condition (face-to-face vs. online), and the interaction of phi and condition** (n=61 groups).

|  | **Step 1** | **Step 2** | **Step 3** |
| --- | --- | --- | --- |
| Phi | 0.645** | 0.645** | 0.644* |
| Condition |  | 0.172 | 0.172 |
| Phi x Condition |  |  | 0.003 |
|  |  |  |  |
| R^2^ | 0.137** | 0.139** | 0.139** |
| R^2^ change |  | 0.002 | 0.000 |

* p <. 05, ** p < .01
