# Supplementary material for: Integrated information as a metric for group interaction
Source: PLoS One. 2018 Oct 11;13(10):e0205335. doi: 10.1371/journal.pone.0205335 (PMC6181355; doi:10.1371/journal.pone.0205335)
Supplement: S5 Table — Descriptive statistics of the number of articles, editors, edits, and edits per editor in various periods before quality changes in the Wikipedia dataset (time windows of 30-, 60-, and 90-days shown in panels A, B, and C, respectively). (DOCX) [file pone.0205335.s009.docx]

**S5 Table**: **Descriptive statistics of the number of articles, editors, edits, and edits per editor in various periods before quality changes in the Wikipedia dataset** (time windows of 30-, 60-, and 90-days shown in panels A, B, and C, respectively).

|  |  | **FA** | **A** | **GA** | **B** | **C** |
| --- | --- | --- | --- | --- | --- | --- |
| Number of articles |  | 136 | 108 | 185 | 535 | 169 |
| Number of | Mean | 16.206 | 12.806 | 10.984 | 9.164 | 6.728 |
| editors per | Min | 2 | 3 | 3 | 2 | 2 |
| article | Max | 72 | 56 | 63 | 51 | 18 |
|  | Std | 12.075 | 9.216 | 8.589 | 7.111 | 3.382 |
| Number of | Mean | 201.544 | 94.241 | 110.676 | 50.206 | 34.817 |
| edits | Min | 4 | 8 | 8 | 4 | 6 |
| per article | Max | 1049 | 861 | 825 | 857 | 318 |
|  | Std | 210.452 | 136.702 | 137.086 | 78.652 | 44.514 |
| Number of | Mean | 12.153 | 8.039 | 10.909 | 5.356 | 5.611 |
| edits per | Min | 1.75 | 1.833 | 1.8 | 1.667 | 1.75 |
| editor per | Max | 72.8 | 172.2 | 71.8 | 159.2 | 58.333 |
| article | Std | 11.186 | 17.157 | 11.857 | 9.422 | 8.765 |

(A) 30-day period before quality change

|  |  | **FA** | **A** | **GA** | **B** | **C** |
| --- | --- | --- | --- | --- | --- | --- |
| Number of articles |  | 138 | 129 | 215 | 699 | 273 |
| Number of | Mean | 25.92 | 20.163 | 16.893 | 13.701 | 9.183 |
| editors | Min | 3 | 3 | 2 | 2 | 2 |
| per article | Max | 115 | 89 | 97 | 77 | 31 |
|  | Std | 19.411 | 16.355 | 13.851 | 11.39 | 5.611 |
| Number of | Mean | 326.768 | 152.24 | 170.995 | 79.495 | 45.388 |
| edits per | Min | 9 | 7 | 8 | 4 | 4 |
| article | Max | 2341 | 1637 | 969 | 1635 | 350 |
|  | Std | 345.283 | 213.7 | 175.6 | 124.957 | 52.16 |
| Number of | Mean | 12.655 | 9.036 | 12.052 | 5.66 | 4.931 |
| edits per | Min | 2 | 1.75 | 2 | 1.75 | 1.75 |
| Editor per | Max | 70.939 | 272.833 | 104 | 272.5 | 47.5 |
| article | Std | 10.908 | 24.181 | 13.133 | 11.846 | 6.19 |

(B) 60-day period before quality change

|  |  | **FA** | **A** | **GA** | **B** | **C** |
| --- | --- | --- | --- | --- | --- | --- |
| Number of articles |  | 139 | 135 | 226 | 773 | 345 |
| Number of | Mean | 34.576 | 26.837 | 22.845 | 18.107 | 11.533 |
| editors | Min | 6 | 4 | 3 | 2 | 2 |
| per article | Max | 152 | 110 | 121 | 123 | 41 |
|  | Std | 26.284 | 20.8 | 18.114 | 15.609 | 8.222 |
| Number of | Mean | 413.281 | 203.541 | 229.615 | 106.585 | 54.939 |
| edits per | Min | 21 | 11 | 9 | 3 | 3 |
| article | Max | 2550 | 1653 | 1721 | 1661 | 420 |
|  | Std | 407.634 | 243.445 | 231.389 | 151.653 | 61.581 |
| Number of | Mean | 12.466 | 9.051 | 12.47 | 5.647 | 4.591 |
| edits per | Min | 2.231 | 2.2 | 1.8 | 1.5 | 1.5 |
| editor per | Max | 67.105 | 236.143 | 245.857 | 237.286 | 46.667 |
| article | Std | 10.067 | 20.515 | 18.921 | 9.684 | 5.049 |

(C) 90-day period before quality change
