# Supplementary material for: Integrated information as a metric for group interaction
Source: PLoS One. 2018 Oct 11;13(10):e0205335. doi: 10.1371/journal.pone.0205335 (PMC6181355; doi:10.1371/journal.pone.0205335)
Supplement: S6 Table — (DOCX) [file pone.0205335.s010.docx]

**S6 Table:** **Regression results when predicting phi for each article from number of edits, average number of edits per editor, and newly acquired quality level of the article**.

|  | **Estimate** | **Std. Error** | **t Value** | **Pr(>\|t\|)** |
| --- | --- | --- | --- | --- |
| (Intercept) | 1.594 | 0.207 | 7.708 | 1.75e-14 *** |
| Number of editors | 0.012 | 0.001 | 18.076 | < 2e-16 *** |
| Edits per editor | -0.123 | 0.009 | -14.46 | < 2e-16 *** |
| Quality B | 0.604 | 0.240 | 2.515 | 0.012 * |
| Quality GA | 0.345 | 0.311 | 1.11 | 0.267 |
| Quality A | 1.291 | 0.359 | 3.592 | 0.0003 *** |
| Quality FA | 0.370 | 0.378 | 0.978 | 0.328 |

Adjusted R-squared = 0.127, F = 70.11, p < 2.2e-16
