# Supplementary material for: Integrated information as a metric for group interaction
Source: PLoS One. 2018 Oct 11;13(10):e0205335. doi: 10.1371/journal.pone.0205335 (PMC6181355; doi:10.1371/journal.pone.0205335)
Supplement: S7 Table — (DOCX) [file pone.0205335.s011.docx]

**S7 Table**: **Number of information packets, origin nodes and destination nodes for each month analyzed.**

| Year | Month | Number of information packets | Number of origin nodes | Number of destination nodes |
| --- | --- | --- | --- | --- |
| 2008 | 8 | 10,419,879 | 284,987 | 248,892 |
| 2009 | 3 | 15,640,702 | 373,440 | 551,702 |
| 2009 | 9 | 17,402,179 | 495,864 | 622,101 |
| 2010 | 3 | 15,322,907 | 343,577 | 399,145 |
| 2010 | 9 | 14,570,116 | 294,594 | 173,678 |
| 2011 | 3 | 18,094,613 | 559,263 | 352,122 |
| 2011 | 9 | 20,132,417 | 411,129 | 412,778 |
| 2012 | 3 | 11,242,632 | 294,890 | 164,138 |
| 2012 | 9 | 10,503,070 | 220,584 | 96,248 |
| 2013 | 3 | 11,195,621 | 1,675,419 | 150,134 |
| 2013 | 9 | 20,739,677 | 274,875 | 204,683 |
| 2014 | 3 | 18,803,582 | 413,410 | 164,051 |

** = p < 10^-6^
