# Supplementary material for: Integrated information as a metric for group interaction
Source: PLoS One. 2018 Oct 11;13(10):e0205335. doi: 10.1371/journal.pone.0205335 (PMC6181355; doi:10.1371/journal.pone.0205335)
Supplement: S8 Table — (DOCX) [file pone.0205335.s012.docx]

**S8 Table**: **Regression coefficients for predicting phi from date with four different node sampling methods while only looking at a fixed number of packets** (10,000,000 packets).

**Node sampling method**

|  | **Random Walk** | **Forest Fire** | **Breadth First** | **Random Nodes** |  |
| --- | --- | --- | --- | --- | --- |
| Regression coefficient | 3.487*** | 3.025*** | 3.850*** | 7.334*** |  |
|  |  |  |  |  | |

*** = p < 10^-6^
